# Unraveling the Role of NeuroD2 in Ischemic Pathophysiology: Insight into Neuroprotection Mechanisms Associated with AKT Survival Kinase

**DOI:** 10.1007/s12017-025-08852-2

**Published:** 2025-04-16

**Authors:** Busenur Bolat, Cigdem Bayraktaroglu, Zehra Degirmenci, Ecem Cerah, Mehmet Sali, Edanur Kolcu, Dila Nur Bars, Cemil Aydin, Fatima Abasova, Abdulla Alisoy, Hasan Ege Atali, Mustafa Caglar Beker, Ulkan Celik, Merve Beker

**Affiliations:** 1 Department of Medical Biology, Institute of Health Sciences, University of Health Sciences Türkiye, Istanbul, Türkiye; 2 Department of Physiology, Institute of Health Sciences, Yeditepe University, Istanbul, Türkiye; 3 Regenerative and Restorative Medicine Research Center (REMER), Research Institute for Health Sciences and Technologies (SABITA), Istanbul Medipol University, Istanbul, Türkiye; 4 International School of Medicine, University of Health Sciences Türkiye, Istanbul, Türkiye; 5 Department of Physiology, School of Medicine, Istanbul Medeniyet University, Istanbul, Türkiye; 6 Department of Medical Biology, School of Medicine, University of Health Sciences Türkiye, Istanbul, Türkiye; 7 Department of Medical Biology, International School of Medicine, University of Health Sciences Türkiye, Mekteb-i Tıbbiye-i Şahane (Hamidiye) Külliyesi Selimiye Mah., Tıbbiye Cad. No: 38, Üsküdar, 34668 Istanbul, Türkiye

**Keywords:** Cell survival, DNA fragmentation, NeuroD2, Oxygen–glucose deprivation, PI3K pathway, Proteomics

## Abstract

NeuroD2 (ND2), a neuron-specific transcription factor, is essential in neural differentiation and neuroplasticity, yet its regulation under neuronal injury is barely uncovered. Effective treatment strategies for ischemic conditions require extensive knowledge of the signaling pathways and mechanisms underlying ischemic pathophysiology. This study aims to uncover the neuroprotective role of ND2 in ischemia and its interactions with critical signaling pathways implicated in recovery. An in vitro ischemic stroke model oxygen–glucose deprivation (OGD) method was applied to neuro-2A (N2a) cells with lentiviral ND2 (LvND2) overexpression. DNA fragmentation and cell survival assays indicated ND2’s neuroprotective and anti-apoptotic effects under OGD conditions. Proteomic profiling and interaction analyses showed that LvND2 regulated the synthesis of cellular signaling, proliferation and cell adhesion-related proteins, such as MAPK3, Mki67, and NCAM. Additionally, a positive correlation was observed between ND2 expression and phosphorylated AKT levels. To investigate the interaction between ND2 and the PI3K/AKT signaling pathway, the pathway was pharmacologically inhibited with Wortmannin 30 min before OGD induction. After 8 h of OGD followed by 16 h of reperfusion, cell survival, DNA fragmentation, and Western blot analyses were performed. LvND2 administration alone increased cellular survival, whereas its combination with Wortmannin resulted in decreased cell survival. Additionally, LvND2 alone reduced the number of TUNEL-positive cells, while its combination with Wortmannin remains non-significant. These findings suggest that ND2 and AKT function in a coordinated manner within the PI3K/AKT survival pathway. ND2 may modulate AKT activity, highlighting its potential as a therapeutic target for addressing ischemic pathophysiology through molecular therapies.

**Graphical Abstract:**

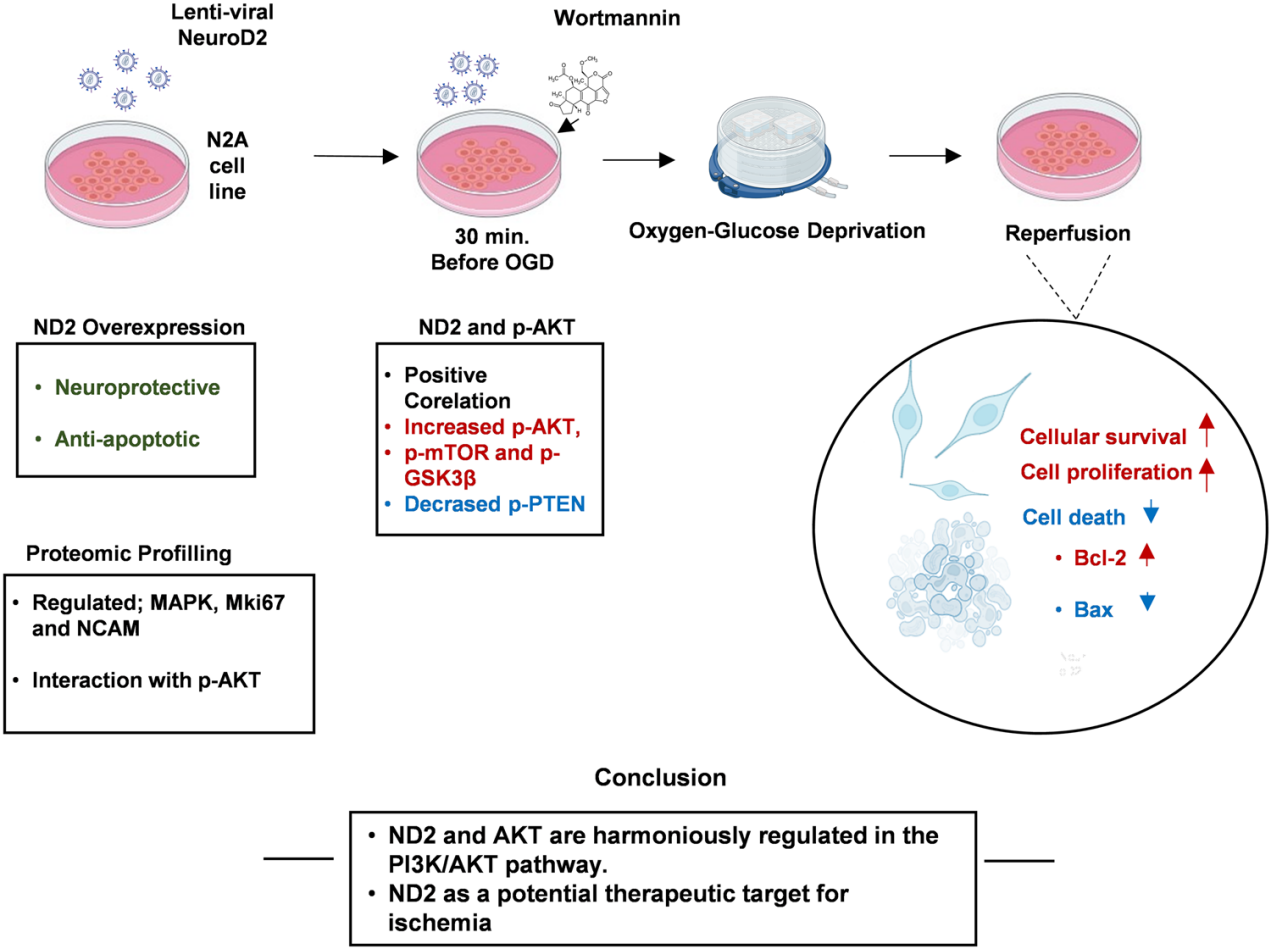

**Supplementary Information:**

The online version contains supplementary material available at 10.1007/s12017-025-08852-2.

## Introduction

Ischemic stroke is a severe, life-threatening condition with devastating consequences (Katan & Luft, 2018; Tsao et al., 2022). Despite extensive research, its pathophysiology remains incompletely understood, and in vivo models pose challenges in exploring its molecular basis (Pignataro et al., 2008). In vitro oxygen–glucose deprivation (OGD) models effectively simulate ischemia–reperfusion by depriving cells of oxygen and glucose, inducing oxidative stress, inflammation, neurotoxicity, pH changes, and cell death mechanisms (Babu et al., 2022; Beker et al., 2019; Sommer, 2017).

Transcription factors (TFs) are vital in the central nervous system, from development to protein regulation (Dennis et al., 2019). Basic helix-loop-helix (bHLH) proteins, a large TF family, regulate neurogenesis, including cell proliferation and differentiation. Subfamilies like HES, OLIG, NPAS, and NEUROD are essential for CNS function (Dennis et al., 2019). NeuroD2 (ND2), expressed in mature, non-dividing neurons, is active in key brain areas such as the cortex and hippocampus (Tutukova et al., 2021). ND2 regulates neurotransmitter fate, axonal plasticity, and synaptic integration (Cardoso et al., 2019; Tutukova et al., 2021). *Olson *et al. demonstrated that deletion of the ND2 gene caused a combination of motor function loss and neurodevelopmental retardation (Olson et al., 2001). ND2 also organizes cortical layers and neuronal migration, with its loss linked to fewer neurons in upper cortical layers (Chen, 2015; Guzelsoy et al., 2019; Ince-Dunn et al., 2006). However, ND2’s molecular mechanisms remain mostly unclear.

For decades, researchers have endeavored to develop effective therapies to mitigate the detrimental consequences of ischemic stroke. Investigations have primarily focused on the protective and regenerative functions of signaling pathways to address ischemic pathophysiology from both molecular and physiological perspectives. The PI3K/AKT pathway, crucial for regulating cell survival, proliferation, and regeneration, is particularly significant. All AKT isoforms are abundant in the nervous system and contribute to neuroprotection (Levenga et al., 2021). PI3K/AKT pathway includes many regulator proteins, including AKT, PTEN, mTOR and GSK3. GSK3 is known as a signal transducer of AKT in PI3K/AKT pathway (Guo et al., 2016). Studies suggest that PI3K/AKT pathway activation after ischemia is vital for neuronal recovery, modulating oxidative stress, inflammation, and related processes (Beker et al., 2019; Tian et al., 2019). *Darshit and Ramanathan* showed that OGD exposure reduces ND2 expression in SH-SY5Y neuroblastoma cells, while GSK-3β inhibitor treatment restores ND2 expression and supports neuronal maintenance (Darshit & Ramanathan, 2016).

Although accumulating evidence points to its versatile roles, ND2 requires further investigation to fully understand its functional actions and interactions with intrinsic signaling proteins. In this study, the Neuro2a (N2a) mouse cell line was transduced with lentiviral ND2 under OGD conditions. Cellular survival, DNA fragmentation, and the expression of p-AKT, p-mTOR, p-PTEN, p-GSK3β, Bcl-2, and Bax were analyzed. LC–MS/MS proteomic analyses revealed that ND2 overexpression alters protein expression during OGD, potentially impacting key pathways like PI3K/AKT and MAPK/ERK. To explore ND2's relationship with AKT, Wortmannin, a PI3K inhibitor, was applied, and its effects on cellular survival and DNA fragmentation were assessed.

## Materials and Methods

### Experimental Design and Groups

**Fig. 1:**
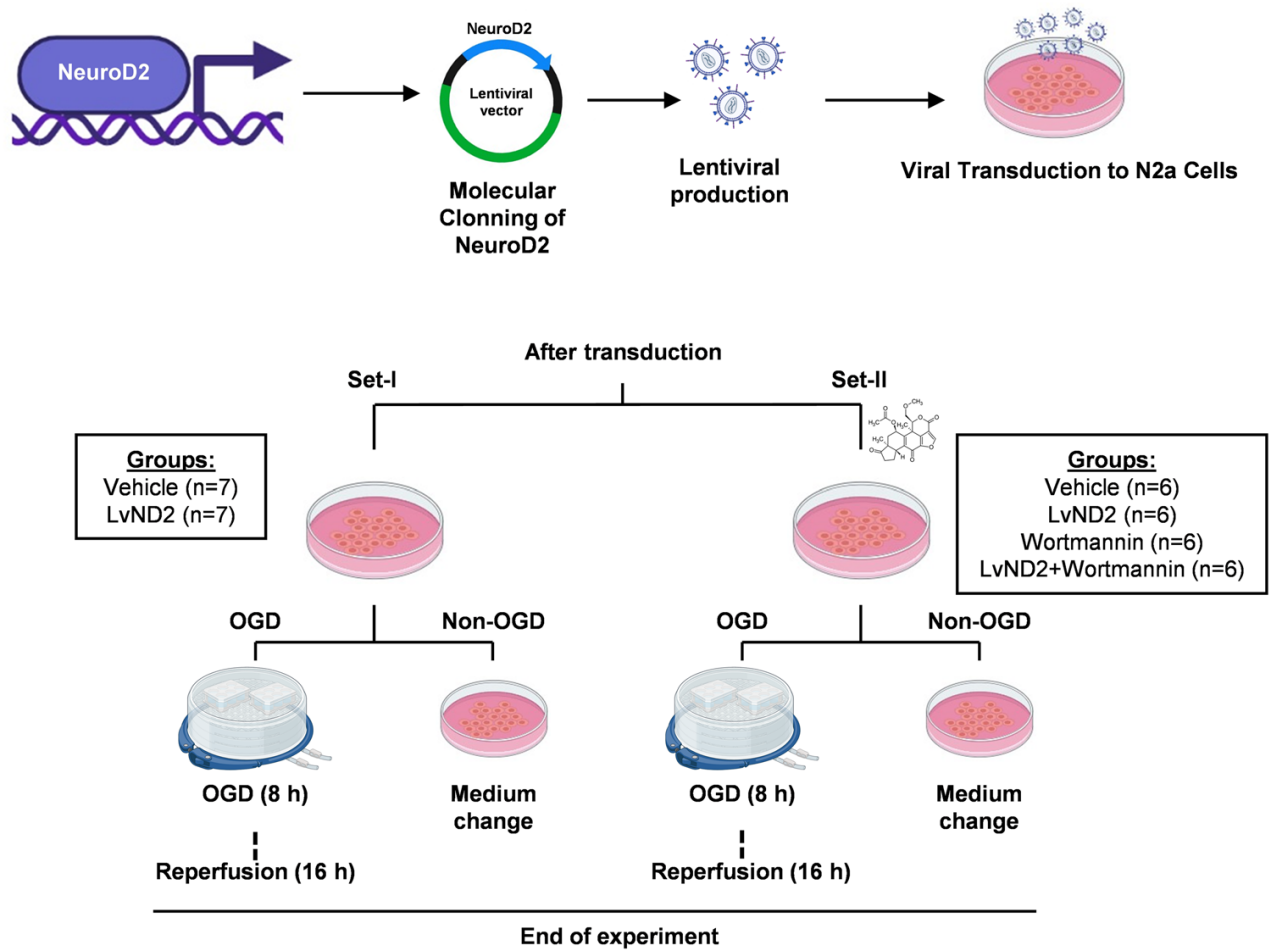
Experimental design. LvND2 viral particles were purified and transduced into Neuro2a (N2a) cells. Twenty-four hours after transduction, the oxygen–glucose deprivation (OGD) model was performed to the cells for 8 h (n = 7 for Set-I and n = 6 for Set-II). Following OGD, reperfusion was performed for 16 h, and then the cells were collected for cell survival assay, TUNEL, Western blot, and proteomics analysis. In the non-OGD (control) set, the medium was changed instead of the OGD application, and other experimental steps were performed in the same way. After all the analyses, depending on the results from the proteomic analysis, another experimental set was created with a PI3K/AKT inhibitor, Wortmannin. After viral transduction, Wortmannin was administered to the membranes of the cells 30 min before OGD treatment. Following OGD for 8 h, reperfusion was performed for 16 h. The medium was changed instead of OGD application in the non-OGD set, and other experimental steps were performed in the same way

The coding sequence of ND2 (NCBI Ref. Seq. NM_010895.3) was cloned into the lentiviral vector (pLenti-EF1a-GFP-2A-Puro, LV067, Applied Biological Materials). Using an empty lentiviral vector (vehicle) and the lentiviral ND2 (LvND2), viral particles were produced, purified, and transduced into Neuro2a (N2a) cells. In the first set of experiments, the cells were subjected to 8 h of oxygen–glucose deprivation (OGD) followed by 16 h of reperfusion. In this set, we evaluated cell survival, DNA fragmentation, and signal transduction pathways using LC–MS/MS and protein expressions elevated with Western Blot (Fig. 1). A second set of experiments was conducted to analyze whether LvND2 acts through the PI3K/AKT signaling pathway. In this set, 1 µM Wortmannin was administered 30 min before the OGD to inhibit the PI3K/AKT signaling pathway. After 30 min, the cells underwent 8 h of OGD followed by 16 h of reperfusion. Cellular survival, DNA fragmentation, and the abundances of ND2, p-AKT, p-mTOR, p-PTEN, and p-GSK3β were determined using Western blot (Fig. 1). In both sets of experiments, no OGD was applied to the control groups (non-OGD). Only medium switching was performed to equalize the experimental conditions.

### Cell Culture

Mouse N2a cells were obtained from the American Type Culture Collection (ATCC) and cultured in low-glucose Dulbecco’s Modified Eagle Medium (DMEM; Pan Biotech, P04-01515) supplemented with 10% Fetal Bovine Serum (FBS; FBS-11A, Capricorn) at 37 °C and 5% CO_2_. Cells were sub-cultured regularly once they reached the required density.

### Molecular Cloning of NeuroD2 and Lentivirus Production

Total RNA was extracted from cultured N2A cells using the Pure-Link RNA mini kit (12183018A, Thermo Fisher Scientific), and cDNA was synthesized using the qScript cDNA Synthesis Kit (95047-100, Quanta). The primers were designed using the ND2 coding sequence (NCBI ref. seq. NM_010895.3) (Forward primer: 5ʹ-AGTCAGAATTCATGCTGACCCGCCTGTTC-3ʹ Reverse primer: 5ʹ-AGTCAGTCGACTCAGTTATGGAAAAATGCGTTGAG-3ʹ). The ND2 coding sequence was amplified via PCR and restricted with fast digest enzymes EcoRI (FD0275, Thermo Fisher Scientific) and SalI (FD0644, Thermo Fisher Scientific) together with the lentiviral vector (pLenti-EF1a-GFP-2A-Puro, LV067, Applied Biological Materials). The gene of interest was ligated into the cloning vector using T4 DNA ligase (EL0014, Thermo Fisher Scientific), and re-digested to confirm the ND2 insert.

A second-generation lentivirus packaging method was used to create vectors according to safety guidelines. pMD2.G (Plasmid #12259, Addgene) and psPAX (Plasmid #12260, Addgene) packaging plasmids were used as complementary vectors for packaging the lentiviral system. HEK293T cells (6 × 10^6^cells) were seeded on 10 cm plates. The transfection process was carried out using Lipofectamine 3000 (L3000015, Thermo Fisher Scientific) to generate DNA-lipid complex. LvND2 (7 μg), pMD2.G (3.5 μg), and psPAX (7 μg) vectors mixed in lipid complex were applied to cells drop by drop. The medium was supplemented with fresh DMEM (Pan biotech, P04-01515) 6 h after transfection, and the cells were incubated at 37 °C in a moist environment containing 5% CO_2_. The whole medium was harvested twenty-four and fifty-two h after transfection, centrifuged for ten minutes at 2000 rpm, and filtered with a low binding filter with a pore size of 0.45 µm. Virus particles were collected after ultracentrifugation at 120,000 g for 2 h and dissolved in Dulbecco’s Phosphate-Buffered Saline (DPBS; P04-3650, Pan Biotech) without calcium and magnesium. As a control, an expression plasmid without the ND2 insert was packaged using the same protocol (Beker et al., 2019, 2020).

For viral transduction, 4 × 10^5^ cells were plated per well in 6-well cell culture plates. The following day, viral transduction was performed by adding the virus particles dropwise. After 24 h, the medium was replaced with fresh DMEM and the cells were incubated at 37 °C in a humidified atmosphere with 5% CO_2_.

### Inhibition of Phosphatidylinositol 3-Kinase/AKT Signaling Pathway

Wortmannin (W1628-1MG, Sigma Aldrich) was used to inhibit the PI3K/AKT signaling pathway during OGD. In each set of experiments, either the vehicle (100% Dimethyl sulfoxide (DMSO)) or 1 µM Wortmannin (dissolved in 100% DMSO) was administered 30 min before OGD (Beker et al., 2019).

### Oxygen–Glucose Deprivation Model (OGD)

OGD was performed 24 h after viral transduction to mimic ischemic conditions. The standard culture medium was replaced with equilibrated (5% CO_2_, 95%N_2_) no-glucose DMEM (11966, Gibco) for OGD. The plates were placed in a hypoxic incubator chamber (27310, Stemcell Technologies) supplemented with a gas mixture of 5% CO_2_ and 94% N_2_. After 8 h at 37 °C, the OGD medium was replaced with fresh low-glucose DMEM supplemented with 10% FBS, and the cells were incubated for 16 h for reperfusion. Following reperfusion, cells were kept for further analyses (Rizzo et al., 2021).

### Cell Survival Analysis

Cells were counted in five different regions of interest (ROI) for both non-OGD and OGD sets. Cell culture plates were washed once with no-glucose DMEM before re-oxygenation to eliminate dead and unattached cells in oxygen and glucose-deprived cells. The mean of surviving cells after OGD was calculated and normalized to the control group (non-OGD vehicle) which transfected with empty lentiviral vector that did not undergo OGD. All cell counting during the in vitro experiments was conducted in a blinded manner (Beker et al., 2019; Stoddart, 2011).

### DNA Fragmentation Analysis

To investigate DNA fragmentation, cells were stained by terminal transferase fluorescein-dUTP nick end labeling (TUNEL) using an in-situ cell death detection kit (1215692910, Roche). Briefly, the entire medium was removed, and the cells were delicately washed three times with DPBS. After that, the cells were fixed with 4% paraformaldehyde (PFA) for 10 min before being washed with DPBS. TUNEL solution was applied to the cells after blocking them with Tris Buffer Saline-Tween20 (TBS-T) for 20 min and then incubated at 37 °C for 1 h. Finally, each well was counterstained with 4′,6-Diamidino-2-Phenylindole, Dihydrochloride (DAPI). TUNEL-positive DNA-fragmented cells were counted blindly from 5 adjacent ROIs using an LSM 880 confocal microscope (Carl Zeiss, Jena, Germany) (Beker et al., 2019).

### Western Blot

Collected cells were centrifuged at 2000×*g* for 10 min at 4 °C, and the supernatant was discarded. The precipitated cells were lysed with lysis buffer (IP lysis buffer; 87788, Thermo Fisher Scientific Scientifics) and phosphatase-protease inhibitor cocktail (5872, Cell Signaling Technologies). Samples were incubated on ice for 15 min and centrifuged at 14,000×*g* for 10 min at 4 °C. After centrifugation, the supernatants were transferred to new tubes. For the preparation of loading samples, protein concentration was determined by Qubit instrument (Q33238, Thermo Fisher Scientific). After that, protein samples were mixed with loading dye with calculated for 20 µg protein for each well for SDS-PAGE gel (1610737, Laemli buffer, Bio-Rad Life Sciences Research). Then samples were loaded onto gels containing 4–20% TGX (Tris-glycine) (4561094, Bio-Rad Life Sciences), run at 150 V for 1 h, and transferred to polyvinylidene fluoride (PVDF) membranes (162-0174, Bio-Rad Life Sciences Research). Following transfer, the membranes were treated with Tris Buffer Saline-Tween20 (TBS-T) containing 5% skimmed milk powder for 1 h at room temperature and incubated overnight with anti-β actin (#643807, Biolegend), anti-ND2 (ab104430, Abcam, USA), anti-p-AKT (#9272s, Cell Signaling Technologies), anti-p-PTEN (#9554s, Cell Signaling Technologies) anti-Bcl-2 (#2876, Cell Signaling Technologies), anti-Bax (#2772, Cell Signaling Technologies), anti-p-MTOR (#2971, Cell Signaling Technologies) and anti-p-GSK3α/β (#8566, Cell Signaling Technologies). All primer antibodies used in 1:1000 dilution. Next day, membranes were rinsed with TBS-T and incubated with anti-rabbit HRP or anti-mouse-HRP secondary antibodies for 1 h. PVDF membranes were developed via Western Bright Sirius ECL solution (K-12043-D10, Advansta) and visualized using the Chemi-Doc MP System (1708280, Bio-Rad; Life Sciences Research). Protein expression levels were evaluated using an image analysis system (Image J; National Institute of Health, Bethesda, MD, USA) and normalized according to β -actin (#643807, Biolegend) levels (Beker et al., 2020).

### Correlation Analysis

Correlation analysis was performed using relative ND2 and p-AKT expressions obtained from independent Western blot experiments. Only data from the Vehicle groups under OGD conditions were included in the analysis to establish a baseline and assess potential associations between ND2 and p-AKT expression. Protein expression levels were quantified via densitometric analysis using ImageJ software (National Institutes of Health, Bethesda, MD, USA), and values were normalized to β-actin as a loading control. Pearson’s correlation analysis was conducted using GraphPad Prism 9 software to evaluate the relationship between ND2 and p-AKT levels. Statistical significance was set at p < 0.05 (Dragowska et al., 2000).

### Sample Preparation for Liquid Chromatography–Mass Spectrometry (LC–MS/MS)

LvND2 and Vehicle groups which were exposed to OGD were used for proteomic analyses. Firstly, proteins were broken down into peptides for proteomic analysis. FASP (Filter Aided Sample Preparation) kit (44250, Expedon) was utilized. According to the manufacturer's recommendations, unrelated chemicals and urine were eliminated from protein samples. Then, 50 µg of protein was treated with 50:1 or 100:1 trypsin enzyme in a 50 mM ammonium bicarbonate solution for 16 h at 37 °C. The following day, samples were centrifuged at 14,000×*g* for 10 min before being lyophilized with lyophilization equipment. Then, these lyophilized peptides were dissolved in 0.1% formic acid and prepared at a final concentration of 100 ng/L (Beker et al., 2019; Hacariz et al., 2014).

### LC–MS/MS Analysis and Data Processing

The LC–MS/MS analysis and the subsequent protein identifications were performed according to a previously published protocol (Beker et al., 2019, 2023; Hacariz et al., 2014). Briefly, samples were analyzed using an ACQUITY UPLC M-Class system coupled to a SYNAPT G2-Si high-definition mass spectrometer (Waters). The columns were equilibrated with 97% mobile phase (0.1% formic acid in LC–MS grade water) at a column temperature of 55 °C. Peptides were separated using a 90-min gradient elution from the ACQUITY UPLC M-Class Symmetry C18 trap column (180 µm × 20 mm; Waters) to the analytic column (ACQUITY UPLC M-Class HSS T3 Column, 100 Å, 1.8 µm, 75 µm × 250 mm; 186007474, Waters) at a flow rate of 0.400 μL/min, with a gradient ranging from 4 to 40% hyper grade acetonitrile (Merck) containing 0.1% formic acid (v/v). Positive ion mode MS and MS/MS scans were performed sequentially with a cycle time of 0.6 s. Low collision energy (CE) was set to 10 V, and high CE to 30 V. Ion mobility separation (IMS) was used, with a wave velocity ramped from 1000 m/s to 55 m/s over the IMS cycle, a mobility trapping release time of 500 μs, trap height set to 15 V, and an IMS wave delay of 1000 μs following trap release. All ions within the 50–1900 m/z range were fragmented in resolution mode without precursor ion preselection. A 100 fmol/μL solution of Glu-1-fibrinopeptide B (186007091-2, Waters MA) was used as a lock mass reference at 60-s intervals to monitor mass stability.

Data were analyzed using Progenesis-QI for proteomics software (Waters) to identify and quantify peptides. All proteins were evaluated with at least 3 unique peptide sequences, and the expression ratio was calculated. The identified protein expressions were determined by heat map analysis. PANTHER software (https://pantherdb.org, Protein Analysis Through Evolutionary Relationships) has been used for protein categorization and molecular function analysis, while STRING software (https://cn.string-db.org, Search Tool for the Retrieval of Interacting Genes/Proteins) was used to perform protein–protein interactions. The level of confidence was set at 0.7, and the fold change value was set at 1.4 The Kyoto Encyclopedia of Genes and Genomes (KEGG), Reactome pathway analysis and Gene Ontology (GO) enrichment was constructed using the DAVID (https://david.ncifcrf.gov, version 6.8). For the statistical analysis of proteomic data, multiple comparison correction was not applied, as this study follows an exploratory approach. We focused on identifying biologically relevant trends rather than strictly controlling for multiple testing (Beker et al., 2019, 2023; Hacariz et al., 2014).

### Statistical Analysis

GraphPad Prism 9 was used to compare statistical data. Unpaired t-tests and a one-way ANOVA were used to compare groups, followed by Welch's adjustments. All values are shown as mean ± standard deviations (SD), and p values of < 0.05 were considered significant throughout the experiment.

## Results

### LvND2 Treatment Increased Cellular Survival Under OGD Conditions

**Fig. 2:**
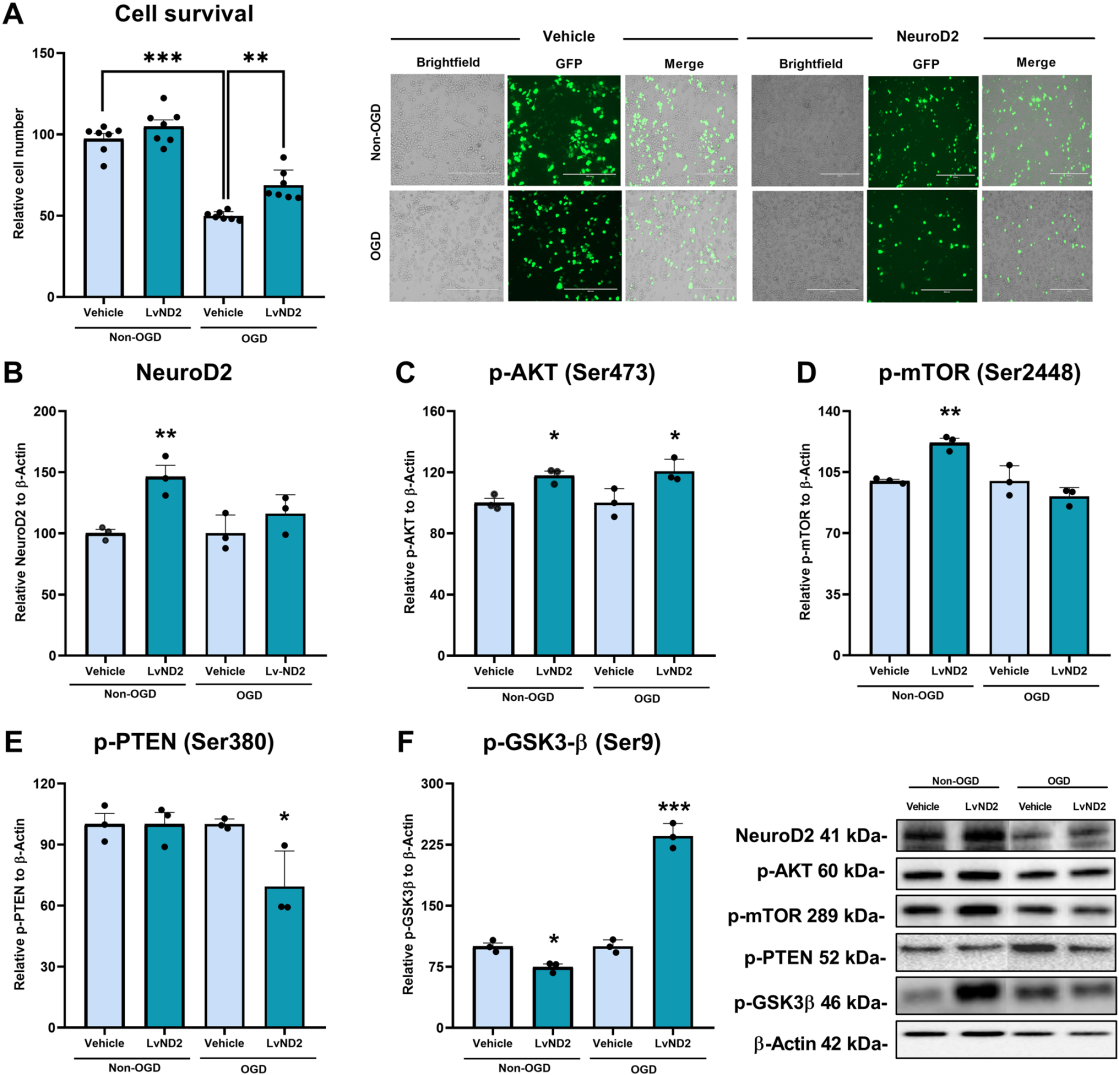
LvND2 overexpression promotes cellular survival and regulates the key proteins of PI3K/AKT pathway under both OGD and non-OGD conditions. **A** In both OGD and non-OGD situations, five randomly chosen regions of interest (ROI) have been identified in order to evaluate the cellular survival of the vehicle and LvND2 groups. The empty viral vector in the vehicle group and the LvND2 viral vector are shown by GFP-positive cells representatively. Significant differences between the vehicle groups in both OGD and control conditions are shown by the validation of the OGD model. In addition, there is a significant rise between the LvND2 and vehicle groups under OGD conditions (***p < 0.001, **p < 0.01). Scale bars represent 400 µm. Western blot analysis was performed to examine PI3K/AKT pathway protein expression under OGD and control conditions in vehicle and LvND2 groups, normalized to β-actin. **B** LvND2 significantly increased NeuroD2 expression under physiological conditions (**p < 0.01), but this effect was not observed under OGD. **C** p-AKT levels increased in the LvND2 group under both conditions (*p < 0.05). **D** p-mTOR expression significantly increased in the LvND2 group under physiological conditions (**p < 0.01) but remained unchanged under OGD. **E** p-PTEN levels were unaffected in non-OGD conditions but decreased in the LvND2 group under OGD (*p < 0.05). **F** p-GSK3β levels significantly decreased in LvND2 under non-OGD conditions (*p < 0.05) but increased under OGD (***p < 0.001)

The effect of LvND2 on cell survival under physiological conditions (control) and after OGD was analyzed. Cell survival assay utilized counting cells in five different regions of interest (ROI) for both control and OGD sets. GFP and brightfield images indicate that viral transduction was carried out successfully. While LvND2 group slightly increased cell number under physiological conditions, the increase was not statistically significant. The comparison of vehicle groups (control cells, treated with an empty Ef1α vector) in both control and OGD conditions, indicates that OGD successfully induced an abrupt cell death (***p < 0.001). Notably, LvND2 significantly enhanced cellular survival compared to the control group under OGD conditions (**p < 0.01) (Fig. 2A).

### Under both OGD and Physiological Conditions, Overexpression of ND2 Regulated the Critical Proteins of the PI3K/AKT Pathway

Under physiological conditions, NeuroD2 (ND2) protein expression significantly increased in the LvND2 group (**p < 0.01), confirming exogenous LvND2 expression. However, ND2 expression remains unsignificant under OGD conditions (Fig. 2B). To explore its interaction with the PI3K/AKT pathway, we analyzed key pathway proteins. p-AKT (Fig. 2C) expression significantly increased in the LvND2 group under both non-OGD and OGD conditions (*p < 0.05). Similarly, p-mTOR levels (Fig. 2D) significantly increased in the LvND2 group under non-OGD conditions (**p < 0.01) but remained unchanged following OGD. Meanwhile, p-PTEN expression (Fig. 2E) remained unaffected under non-OGD conditions but significantly decreased in the LvND2 group after OGD (*p < 0.05).

In contrast, p-GSK3β expression (Fig. 2F) significantly decreased in the LvND2 group under non-OGD conditions (*p < 0.05) but dramatically increased following OGD (***p < 0.001). These findings suggest that while ND2 overexpression enhances AKT activation, its regulatory effects on downstream proteins vary depending on oxygen availability.

### LvND2 Reduced DNA Breaks Associated with Apoptosis and Modulated the Expression of Cell Death-Related Proteins

**Fig. 3:**
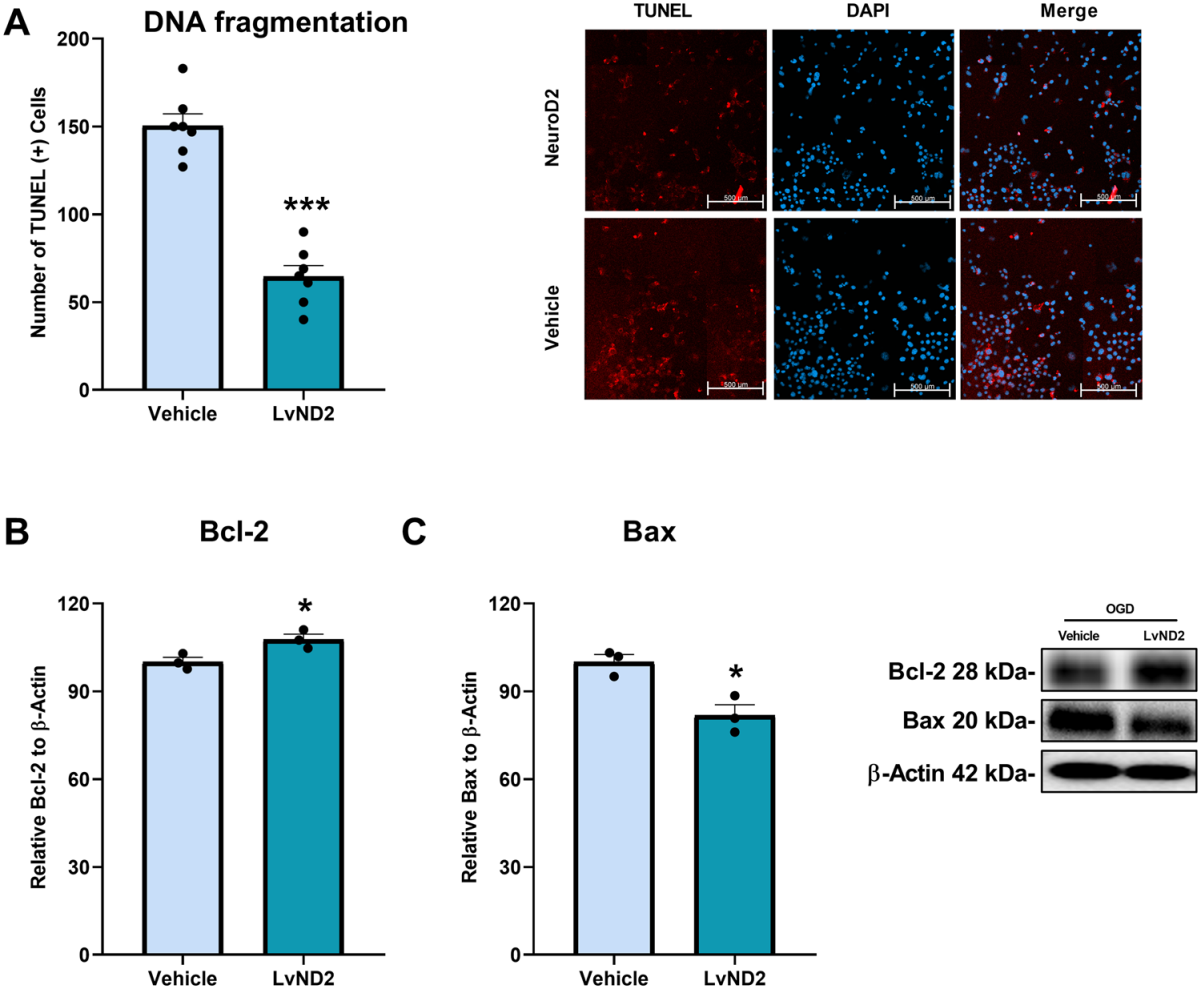
Under OGD conditions LvND2 treatment regulates apoptosis-related protein expressions and decreases TUNEL-positive cell numbers. **A** The TUNEL assay was applied to identify DNA breaks caused by OGD. Five randomly selected regions of interest (ROI) were selected and analyzed for the vehicle and LvND2 groups. Notably, LvND2 significantly decreased TUNEL-positive cells according to the vehicle (***p < 0.001). Scale bars represent 500 µm. **B** Bcl-2 and **C** Bax proteins expressions are examined between the vehicle and LvND2 groups under OGD conditions via Western blot. Bcl-2 protein expressions are increased significantly in the LvND2 group (*p < 0.05). Meanwhile, Bax protein expression levels decreased significantly in the LvND2 group (*p < 0.05)

It is well-known that OGD causes DNA fragmentation. In the current study, we performed terminal deoxynucleotidyl transferase dUTP nick end labeling (TUNEL) staining to determine the effect of ND2 overexpression on DNA fragmentation after OGD and analyzed five random regions of interest (ROI). The results demonstrated that LvND2 significantly reduced the number of TUNEL-positive cells (Fig. 3A) (***p < 0.001). Additionally, we analyzed the expressions of Bax and Bcl-2, two well-known pro- and anti-apoptotic proteins, via Western blotting. Expression of Bcl-2 exhibited a significant increase, consistent with ND2 overexpression (Fig. 3B) (*p < 0.05). Conversely, expression of Bax, whose activation induces apoptotic cell death, showed a significant reduction (*p < 0.05) (Fig. 3C).

### ND2 was a Critical Regulator for Post-OGD Recovery Mechanisms and Played Substantial Roles in Various Biological Processes

Proteomics is widely used to understand disease mechanisms, biomarker discovery, identification of drug targets, analysis of post-translational modifications, and analysis of cell signaling. Unlike gene expression analysis, proteomic analysis offers a more comprehensive biological view by evaluating actual functional proteins and their modifications. It is also a highly innovative and seminal method for proteins whose signaling and interaction mechanisms have not yet been elucidated. Since ND2 protein interactions and signaling mechanisms are not yet enlightened, a proteomic approach was used in this study.

**Fig. 4:**
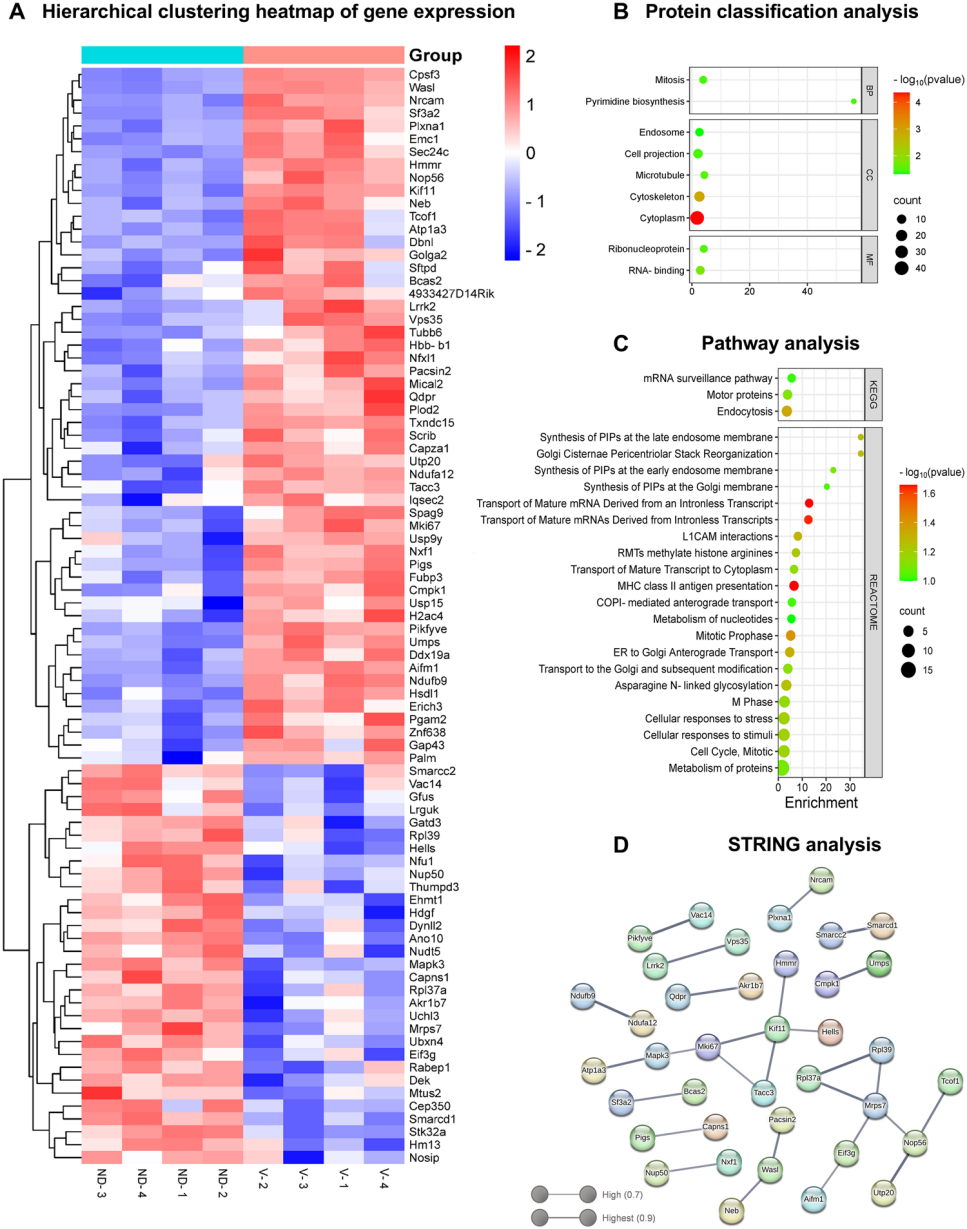
Proteome profiling distinguishes viral ND2 treatment response in OGD-induced N2a cells. **A** The heatmap was created according to the -log 10 p-value obtained from the t-test and was plotted against the log twofold change for each protein that was differently expressed in the LvND2 group. Under OGD conditions, red color represents higher protein levels, and blue color indicates lower levels in the LvND2 group. **B** Using PANTHER software, proteins classified according to (BP), cellular component (CC) and molecular functions (MF). The results of gene ontology enrichment analysis via PANTHER software are displayed in bubble plot as the count of proteins. **C** ND2's KEGG and REACTOME Pathway Enrichments. The pathways are on the Y axis, while the enrichment score is on the X axis. Node color and node area showed positive correlations with the enrichment analysis score and the number of genes expressed. **D** The STRING protein–protein interaction network comprises significantly increased and reduced proteins selected in addition to the log (p-value) > 1.4 cutoff based on a > 1.4-fold change cutoff. After inspecting two distinct modules, a confidence level of 0.7 had been set. Statistical significance was determined without correction for multiple comparisons, as this study follows an exploratory proteomics approach

According to proteomic data, a total of 2020 proteins were identified in both the vehicle and LvND2 groups. Our analyses demonstrated that 85 different proteins significantly (p < 0.05 and a fold change > 1.4) altered, and these proteins were visualized as a heat map using the online SRplot program (available at https://www.bioinformatics.com.cn/) (Fig. 4A) (Tang et al., 2023). Additionally, we performed classification analysis using Protein ANalysis THrough Evolutionary Relationships (PANTHER, http://pantherdb.org) to determine the biological activities of the regulated proteins. Based on gene ontology analysis, the identified proteins were categorized into biological process (BP), cellular component (CC), and molecular function (MF) classifications and visualized as a bubble plot (Fig. 4B). The complete list of gene names along with their corresponding p values and the Volcano plot are provided in Supplementary File 1 and Supplementary Figure 2, respectively.

Additionally, to determine the pathways connected to ND2, Kyoto Encyclopedia of Genes and Genomes (KEGG) and Reactome pathway enrichment analyses were performed via The Database for Annotation, Visualization, and Integrated Discovery (DAVID) (v2023q4, https://david.ncifcrf.gov/). We performed enrichment analyses using the Reactome and KEGG pathways. Pathways ranked along the y-axis display enrichment scores on the x-axis. The balloons' colors indicate statistical significance, which is based on -log10(p-value); results which are closer to red indicate more significance. The number of genes engaging in each pathway is indicated by the size of the balloons. Based on the information collected, these pathways include the following: ER to Golgi Anterograde Transport, mRNA Surveillance Pathway, Motor Proteins, Endocytosis, Golgi Cisternae Pericentriolar Stack Reorganization, Transport of Mature mRNAs Derived from Intron less Transcripts, and Mitotic pathways (Fig. 4C). Subsequently, we used STRING software to investigate protein–protein interactions with a high confidence score (0.7) (Fig. 4D). The STRING analysis identified key proteins like MAPK3 (Mitogen-Activated Protein Kinase 3, also known as ERK-1), NRCAM (Neuronal cell adhesion molecule) and MKI67 (Proliferation marker protein Ki-67), which exhibited relatively higher interactions with other proteins and pathways.

### ND2 Overexpression Increased AKT Phosphorylation in Physiological Conditions

**Fig. 5:**
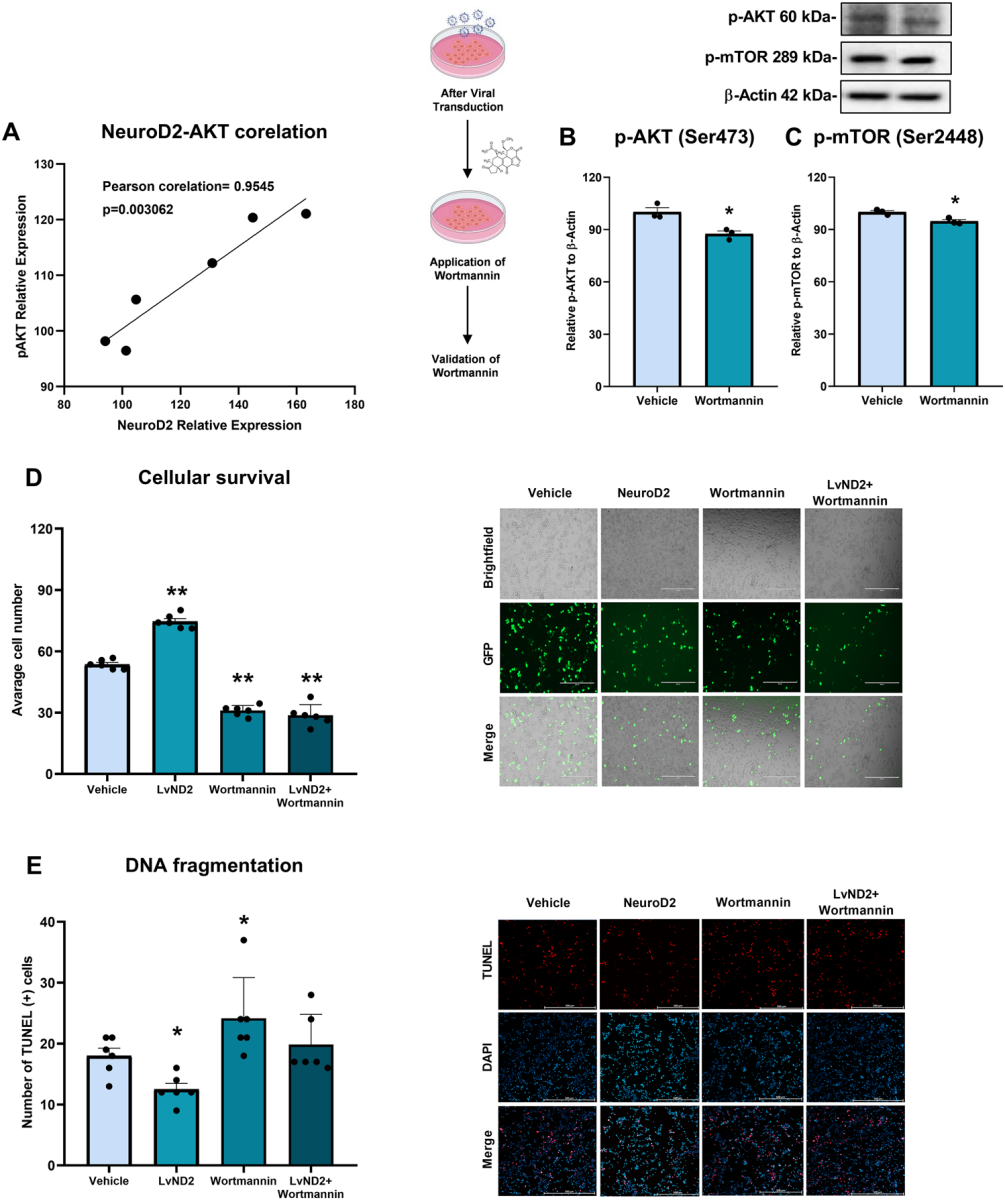
Positive correlations have been discovered between AKT and ND2 proteins and ND2 increases cellular survival and reduces DNA breaks via activating the AKT-PI3K survival pathway. **A** Pearson correlation analysis revealed a significant correlation between NeuroD2 and p-AKT expression (**p < 0.01). Following viral transduction, Wortmannin treatment was validated via Western blot by assessing p-AKT and p-mTOR levels. **B** and **C** Both p-AKT and p-mTOR levels significantly decreased compared to the vehicle group (*p < 0.05). **D** A cell survival assay using five randomly selected ROIs showed that LvND2 increased survival, while Wortmannin and LvND2 + Wortmannin significantly reduced it (**p < 0.01). Scale bars represent 400 µm in each figure. **E** The TUNEL assay indicated reduced DNA breaks only in the LvND2 group, whereas TUNEL-positive cells significantly increased in the Wortmannin and LvND2 + Wortmannin groups (*p < 0.05). Scale bars represent 500 µm in each figure

As ND2 and phosphorylated AKT levels were concomitantly higher in LvND2 group, we questioned whether there was a correlation between ND2 and phosphorylated AKT. Pearson correlation analysis pointed to a significant positive correlation between ND2 relative expression and p-AKT relative expression (Pearson correlation: 0.9545; p = 0.003062) (Fig. 5A).

### ND2 Overexpression Exhibited Neuroprotection Under Oxygen–Glucose Deprivation Conditions But Did Not Reverse the Adverse Effects of PI3K/AKT Pathway Inhibition on Cell Survival

Following the positive correlation observed between ND2 and p-AKT, we further explored the relationship between ND2 and the PI3K/AKT signaling pathway. Initially, we assessed the effect of a 1 µM dose of Wortmannin on p-AKT and p-mTOR protein expressions under physiological conditions using Western blot. The results demonstrated that 1 µM Wortmannin significantly decreased the levels of both p-AKT and p-mTOR protein expressions (* p < 0.05) (Fig. 5B, C). To investigate more about how ND2 and the PI3K signaling pathway work together, we put cells into four groups and applied OGD for 8 h and reperfusion for 16 h. The groups were called Vehicle, LvND2, Wortmannin, and LvND2 + Wortmannin. After that, we examined the effects of these treatments on cell survival and DNA fragmentation. As expected, the LvND2 group by itself exhibited an increased cellular survival, while the Wortmannin group significantly reduced cell survival. Notably, the combination of LvND2 and Wortmannin did not reverse the detrimental effects of Wortmannin on cell survival (**p < 0.01) (Fig. 5D). In contrast, TUNEL assay results showed a significant reduction in TUNEL-positive cells within the LvND2 group (*p < 0.05). Wortmannin aggravated OGD-induced cellular damage, leading to increased cell death, consistent with the survival rate findings (*p < 0.05). Although LvND2 by itself substantially reduced DNA fragmentation, it was unable to fully counteract the effects of Wortmannin in the TUNEL assay (Fig. 5E).

### Upon Exposure to OGD, ND2 Regulated the Expression of Key Proteins in the PI3K/AKT Pathway

**Fig. 6:**
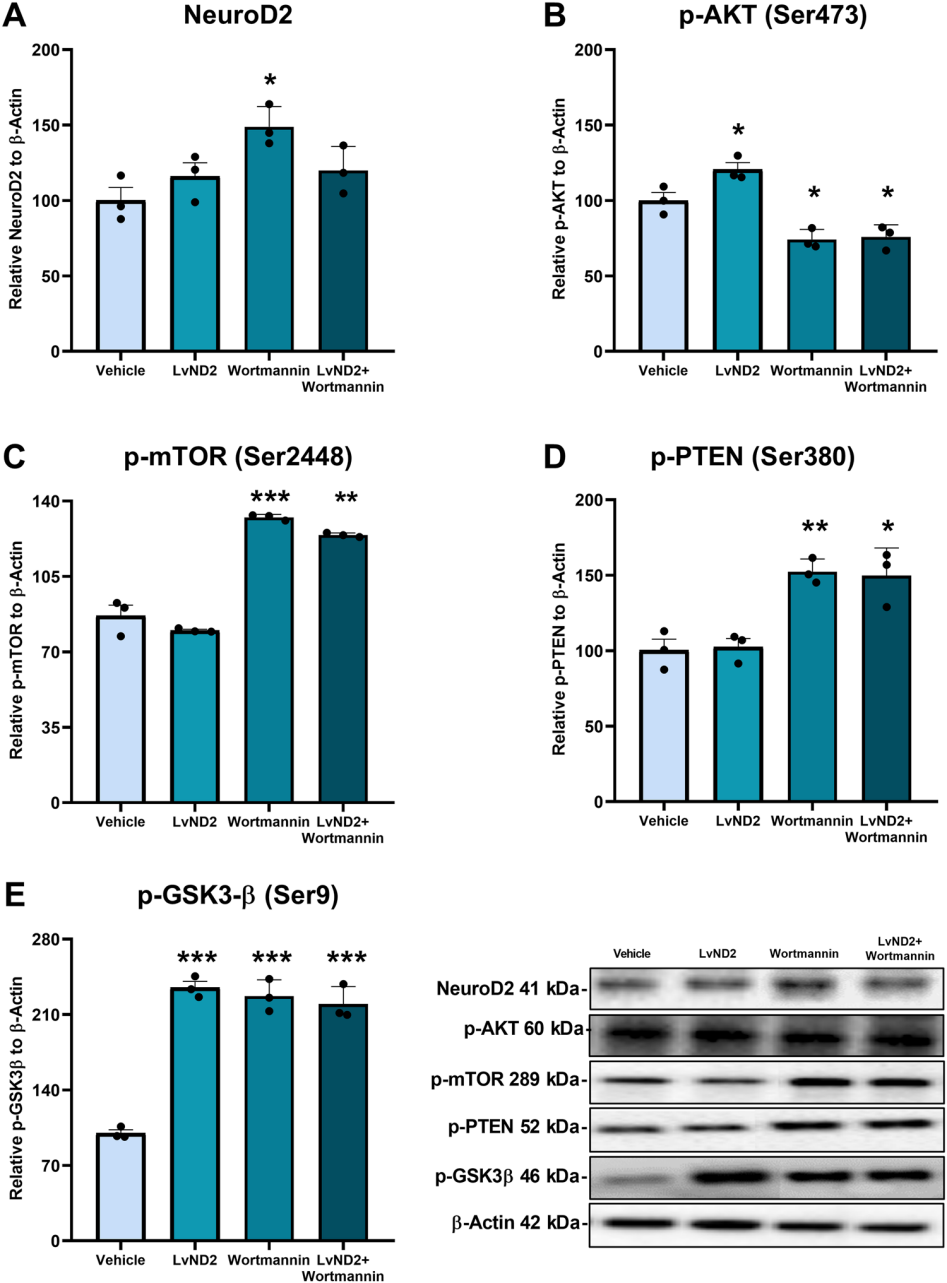
Under OGD conditions, LvND2 regulates essential proteins of PI3K/AKT survival pathway. The relationship between the PI3K/AKT pathway and NeuroD2 (ND2) examined by analyzing p-AKT, p-mTOR, p-PTEN, and p-GSK3β protein expressions, normalized to β-Actin, under OGD conditions. Western blot results showed: **A** NeuroD2 expression significantly increased in the Wortmannin group vs. the vehicle (*p < 0.05). **B** Only the LvND2 group had elevated p-AKT, while Wortmannin and LvND2 + Wortmannin groups showed decreased p-AKT (*p < 0.05). **C** p-mTOR levels significantly increased in Wortmannin and LvND2 + Wortmannin groups (***p < 0.001, **p < 0.01). **D** p-PTEN expression increased in Wortmannin and LvND2 + Wortmannin groups (**p < 0.01, *p < 0.05). **E** p-GSK3β levels increased in LvND2, Wortmannin, and LvND2 + Wortmannin groups (***p < 0.001)

In addition to these findings, we further investigated the components of the PI3K/AKT pathway in relation to ND2 under OGD and Wortmannin treatment. First, we assessed ND2 protein expression levels. Under OGD conditions, the LvND2 group exhibited an upward trend in ND2 expression, although this increase was not statistically significant. Similarly, in Fig. 2B ND2 expression levels showed same expression level under OGD conditions. Interestingly, a significant increase in ND2 protein expression was observed in the Wortmannin group (*p < 0.05) (Fig. 6A). When we examined p-AKT expression levels, the LvND2 group showed a significant increase, consistent with previous results which is showed in Fig. 2C (*p < 0.05). However, both the Wortmannin and LvND2 + Wortmannin groups demonstrated a significant reduction in p-AKT levels (*p < 0.05) (Fig. 6B). Subsequently, we evaluated p-mTOR protein levels. Only the Wortmannin and LvND2 + Wortmannin groups exhibited a significantly increased in p-mTOR levels compared to the vehicle group (***p < 0.001, **p < 0.01) (Fig. 6C). Additionally, we observed a significant rise in the levels of p-PTEN, a negative regulator of AKT, in both the Wortmannin and LvND2 + Wortmannin groups (**p < 0.01, *p < 0.05) (Fig. 6D). Interestingly, p-PTEN expressions showed as decreased in LvND2 group under OGD conditions, but we observed in Fig. 6D the expression changing remains unsignificant. Finally, we assessed p-GSK3β levels and found that all experimental groups, except the vehicle group, showed significantly higher levels of p-GSK3β consistent with previous results which is showed in Fig. 2F (***p < 0.001) (Fig. 6E).

## Discussion

Over time, studies have revealed the functions of ND2 and provided insights into its structure (Bayam et al., 2015; McCormick et al., 1996; Messmer et al., 2012; Olson et al., 2001; Wilke et al., 2012). However, the relationship between ND2 and signal pathways has been the focus of only a handful of studies (Guner et al., 2017; Ince-Dunn et al., 2006; Olson et al., 2001). Our study was designed to discover ND2 as a molecular component of important signaling pathways like PI3K/AKT. In this research, N2a cell line was utilized for developing a model without any differentiation procedures. N2a cells have provided great advantages for mechanism-based approaches in neuroscience. Whereas N2a cells are undifferentiated, they keep the molecular signatures of neurons and have a great potential to differentiate (Beker et al., 2019; Kim et al., 2022).

We investigated the effects of ND2 overexpression in N2a cells under both physiological and OGD conditions. LvND2 significantly regulated survival pathway proteins, including p-AKT, p-mTOR, p-PTEN, and p-GSK3β, under both physiological and OGD conditions (Fig. 2B–F). LvND2 treatment significantly increased ND2 protein expression under physiological conditions compared to the vehicle group. However, under OGD conditions, this increase was not statistically significant, but a rising trend was observed. It has been shown in our cloning studies that the increase in ND2 protein levels under physiological conditions correlates with a parallel rise in p-AKT, suggesting a potential interaction between these proteins. ND2 overexpression continues to increase ND2 protein levels after OGD, although this increase is not statistically significant compared to pre-OGD levels. This may be attributed to cellular stress induced by OGD, which can affect protein expression at both transcriptional and translational levels. Despite the lack of statistical significance, the increase in p-AKT protein expression after OGD remains consistent with the trend observed for ND2 expression. Overall, ND2 and p-AKT seem to be regulated in a similar manner under physiological conditions, but OGD may have an impact on this interaction (Fig. 2B).

Given ND2’s role in survival dynamics, we further examined its influence on cell death. ND2 over-expression positively correlated with Bcl-2 and negatively with Bax protein levels, consistent with reduced TUNEL-positive cells under OGD when LvND2 was administered (Fig. 3A–C). These findings suggest that ND2 acts as a regulatory and neuroprotective factor during OGD, potentially contributing to the PI3K/AKT pathway. To validate this hypothesis, we employed proteomic analysis to identify ND2-coordinated proteins and their associated signaling pathways.

Proteomic profiling showed that ND2 overexpression altered 85 proteins, enhancing 31 and reducing 54, which are related to transporter, catalytic, binding, cytoskeleton motor, and ATP-dependent activities (Fig. 4A–D). Among these proteins the most remarkable ones were Mki67 and Nrcam whose expressions were found to be lower in ND2 transduced cells. Mki-67 (marker of proliferation Kiel 67) is a protein found in actively dividing cells and is commonly used as a marker for cell proliferation. A drop in Mki67 levels in vitro typically indicates a decrease in cell proliferation (Uxa et al., 2021). However, one should also consider that reduced Mki-67 levels might suggest that these cells would have been differentiating. In our research we observed that neuroblastoma cells gave clues of some morphological transformations after ND2 overexpression (Supplementary Fig. 1). It is worth noting that due to its regulatory activities ND2 could be used as an inductor agent for gaining neuroblasts a neuronal identity (Mie et al., 2003; Sugimoto et al., 2009). Consistent with Mki-67, while progressing to later stages of differentiation, LvND2 transfected cells are likely to inactivate certain cell adhesion molecules like Nrcam. Thus, it is reasonable to conclude that ND2 potentially guided survival, proliferation and differentiation mechanisms together. Of further interest Mapk3 (Mitogen activated protein kinase 3) which is also important in developmental contexts was shown to be higher when the cells were treated with ND2 particles. This finding may highlight the crosstalk between Mapk and AKT pathways. At this very point, we conducted a KEGG and REACTOME pathway analysis to investigate-related pathways with ND2 overexpression. Pathway enrichment analysis indicated that ND2, functioning as a neuronal transcription factor, plays roles in stress response, mRNA maturation, and survival processes. This study suggests that either OGD stress or LvND2 transduction could activate the pathways involved. OGD can cause hypoxic stress, which can start signaling pathways that help cells and mRNA stay alive. Our results show that ND2 is linked to the PI3K/AKT signaling pathway. This means that LvND2 transduction could also affect other signaling pathways. In this context, ND2 overexpression could potentially trigger other signaling pathways.

We demonstrated that ND2 is neuroprotective under hypoxia/reoxygenation conditions and regulates key proteins of the PI3K/AKT pathway. Also, hypothesized that ND2 might directly associate with the survival pathway, similar to ND1, another bHLH and NeuroD family member (Jahan et al., 2010). ND1 has been shown to play a critical role in neuronal development and engage in pathways such as PI3K/AKT, WNT, and JNK. Notably, Ma et al. (2022) reported that ND1 protein levels, alongside ND2 and ND6, positively correlated with the activation of these pathways (Ma et al., 2022). Supporting this hypothesis, our findings revealed that LvND2-induced AKT phosphorylation under physiological conditions, suggesting a direct, positive interaction between AKT and ND2. To further explore this relationship, we decided to use pharmacological PI3K inhibitor, Wortmannin. Wortmannin binds to the subunit of PI3K, enters the ATP binding site of the enzyme and blocks the phosphorylation activity of PI3K. Thus, it prevents the conversion of PIP2 to PIP3 and eventually inhibits PI3K/AKT pathway. Also, Wortmannin is common inhibitor which is used in previous studies (Beker et al., 2019). Considering all of this information from literature, we decided to investigate whether ND2 expression on the Wortmannin-applied cells would reverse the inhibitory effects of Wortmannin. According to our data, ND2 acted differently when Wortmannin inhibited PI3K specifically. Instead of reversing the inhibitory effect of Wortmannin, ND2 seemed to act as a neutral element. This domination of Wortmannin on the PI3K/AKT pathway suggests that ND2 may be either placed upstream of AKT or it may activate AKT through other mechanisms than PI3K. To answer these questions, we examined the ND2 protein expression levels (Fig. 6A). The results revealed that neither the combined use of LvND2 + Wortmannin nor LvND2 treatment increased ND2 expression under OGD conditions. It's possible that the cells' exposure to both Wortmannin and OGD prevented the ND2 levels from rising significantly.

Interestingly, Wortmannin increased ND2 expression even without genetic manipulation (Fig. 6A). This effect can be attributed to the activation of other signaling pathways interacting with PI3K/AKT, such as GSK3α/β and MAPK, which are key regulators of ND2 and fine-tune PI3K/AKT signaling (Darshit & Ramanathan, 2016; Lopez-Tobon et al., 2019). Additionally, AKT inhibition may trigger compensatory mechanisms, leading to increased ND2 expression. Wortmannin’s PI3K inhibition likely indirectly influences ND2-related pathways, aligning with prior findings that GSK3β inhibition promotes ND2 expression (Darshit & Ramanathan, 2016). These results support the hypothesis that PI3K/AKT pathway alterations impact ND2 activity, providing evidence of crosstalk between these pathways. Given ND2's involvement in various neuronal functions, the observed increase in ND2 expression under Wortmannin treatment suggests that cells may utilize ND2 as a compensatory mechanism during PI3K/AKT inhibition.

To further explore the pathophysiology of OGD, we investigated ND2 dynamics alongside PI3K/AKT pathway inhibition. Under OGD conditions, expected ND2 expression was absent, likely due to severe disruptions in cellular mechanisms. Surviving cells adapt through transcriptomic and proteomic rearrangements, regulated by transcription factors like HIF1α and CREB (Guo et al., 2018). Interestingly, ND2 expression increased only when OGD was combined with Wortmannin, suggesting this response may counteract cell death induced by hypoxic stress, glucose deprivation, and AKT inhibition. Further studies are essential to unravel the molecular interplay between PI3K and ND2.

We investigated the link between ND2 and AKT by analyzing phosphorylation in the PI3K/AKT pathway (Fig. 6B). ND2 positively regulates AKT under physiological conditions, but PI3K inhibition by Wortmannin suggests a feedback mechanism, as shown by increased p-mTOR levels despite PI3K inhibition (Fig. 6C). Wortmannin may indirectly activate mTOR through stress pathways (Manning & Cantley, 2007). Elevated phosphorylation at PTEN's Ser380/Thr382/383 sites under Wortmannin, regardless of ND2, indicates feedback inhibition of PTEN by reduced AKT activity (Fig. 6D) (Mukherjee et al., 2021). GSK3β, an AKT target, showed regulation beyond PI3K-AKT, being involved in multiple pathways, with roles in neurogenesis, apoptosis, and hypoxia (Marwarha et al., 2022). Our results suggest ND2 may indirectly impact GSK3β based on cellular context (Fig. 6E).

Accumulating evidence highlights the PI3K/AKT pathway as essential for ischemic pathophysiology, as it regulates survival and promotes tissue regeneration (Beker et al., 2019; Li et al., 2022). Numerous in vitro and in vivo studies indicate that AKT activation enhances cell survival mechanisms by inhibiting apoptosis, reducing inflammation, adjusting cellular metabolism, and promoting trophic factor expression (Zheng et al., 2017; Zhou et al., 2015).

In our study, AKT activation was closely associated with ND2 expression (Fig. 5A), highlighting ND2's role as a potential convergence point for pathways like GSK3β, mTOR, and MAPK. ChIP-Seq analysis by *Bayam *et al. identified ND2 binding sites on genome regions encoding Akt3, Fgfr1, and Creb5, key components of the PI3K/AKT pathway with promoters near ND2 motifs (Bayam et al., 2015) (Supplementary Table 1).

This study is a preliminary step towards understanding cellular mechanisms between ND2 and AKT. Our findings suggest a positive correlation between ND2 and AKT through multiple pathways. The main limitation of this study is that the N2a cell line is a proliferative cell, so it would not show the OGD's cellular and metabolic effects properly. Although abrupt metabolic changes during OGD might obscure these interactions, ND2 overexpression demonstrated neuroprotective effects by modulating p-AKT, p-GSK3β, p-mTOR, p-PTEN, and related proteins. However, this study is limited by its in vitro nature, and the complex interactions between ND2 and AKT-associated pathways under physiological conditions remain to be fully elucidated. To validate these findings and explore their translational potential, further studies using animal and primer culture models are essential. Such studies could provide deeper insights into ND2's role in neuroprotection and its therapeutic implications in ischemic conditions.

## Conclusion

In conclusion, this study delved into the neuroprotective mechanisms of ND2 in ischemic conditions, particularly highlighting its pivotal role in activating the PI3K/AKT pathway. Through a comprehensive analysis involving gene ontology studies, protein–protein interactions, and functional assays, we elucidated the diverse activities of ND2 in promoting cell survival and counteracting apoptotic pathways. Moreover, we extended beyond the PI3K/AKT axis and shed light on the intricate network of regulatory pathways orchestrated by ND2 to safeguard neuronal integrity under ischemic challenges. This approach not only enhanced our understanding of ND2 mediated neuroprotection but also underscored its potential as a therapeutic target for mitigating ischemic injury. From this perspective, this study will be of great interest to future studies. Further research would be necessary to elucidate possible specific interactions between AKT and ND2 and to understand the implications of such interactions in cellular processes involving neuronal development and differentiation.

## Supplementary Information

Below is the link to the electronic supplementary material.

Supplementary file1 (XLSX 17 kb)

Supplementary Fig.1: Lentiviral ND2 Application Induces Differentiation in Neuro-2a Cells. A) Vehicle group transfected with an empty lentiviral vector (EF1α) exhibits no signs of differentiation. (PDF 270 kb)

Supplementary Fig.1: Lentiviral ND2 Application Induces Differentiation in Neuro-2a Cells. B) LvND2 group transfected with a NeuroD2 overexpression construct shows clear signs of differentiation. (PDF 269 kb)

Supplementary Fig.2: Volcano plot. The log twofold change for every protein that was differentially expressed in the LvND2 group was plotted against the negative log 10 p-values calculated using the t-test in a volcano plot. The red color represents elevated proteins in the LvND2 group, while the blue color represents decreased proteins. (TIF 643 kb)

Supplementary Table 1: ND2 Target Genes Involved in the AKT-PI3K Survival Pathway. ND2 binding sites within genomic regions, as identified by Bayam et al., (2015), were analyzed to reveal the top 15 proteins with the highest normalized closest gene scores. These proteins are implicated in the AKT-PI3K survival pathway. (PDF 105 kb)

## Data Availability

The raw data from this study are available from the corresponding authors upon reasonable request.
