# Supplementary figures and images for: Unraveling the Role of NeuroD2 in Ischemic Pathophysiology: Insight into Neuroprotection Mechanisms Associated with AKT Survival Kinase
Source: Neuromolecular Med. 2025 Apr 16;27(1):28. doi: 10.1007/s12017-025-08852-2 (PMC12003519; doi:10.1007/s12017-025-08852-2)

Vehicle

A

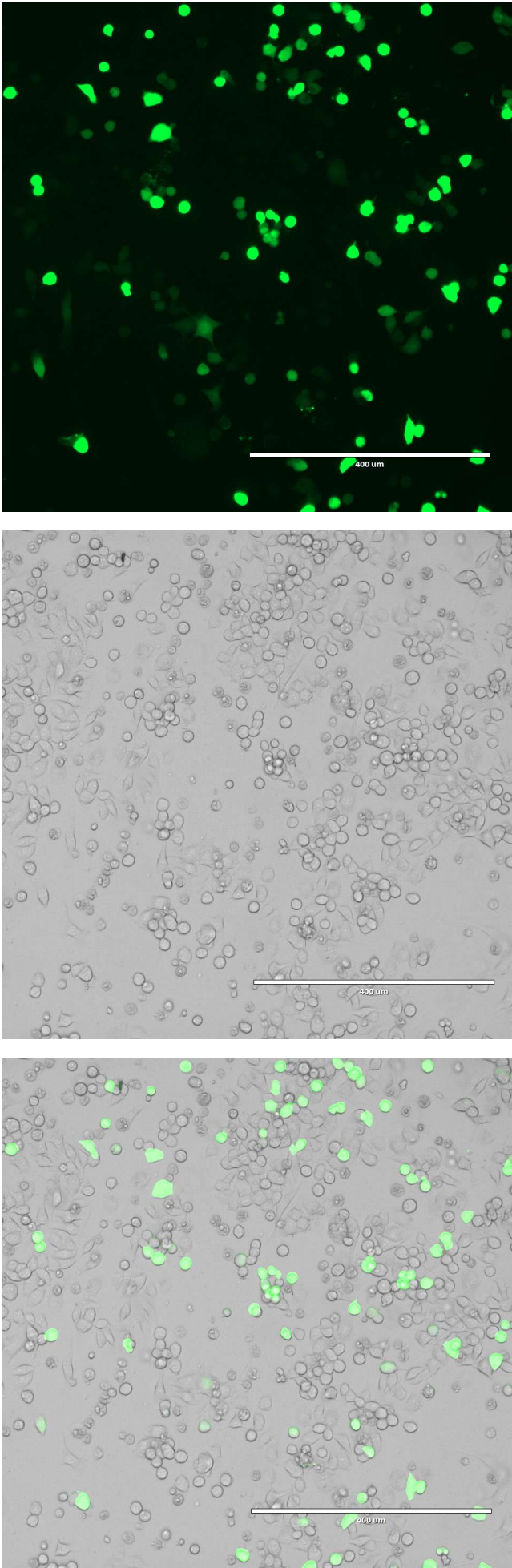

Supplementary fig.1

Supplement: Supplementary file 2 — Supplementary Fig.1: Lentiviral ND2 Application Induces Differentiation in Neuro-2a Cells. A) Vehicle group transfected with an empty lentiviral vector (EF1α) exhibits no signs of differentiation. (PDF 270 kb) [file 12017_2025_8852_MOESM2_ESM.pdf]

# LvND2

B

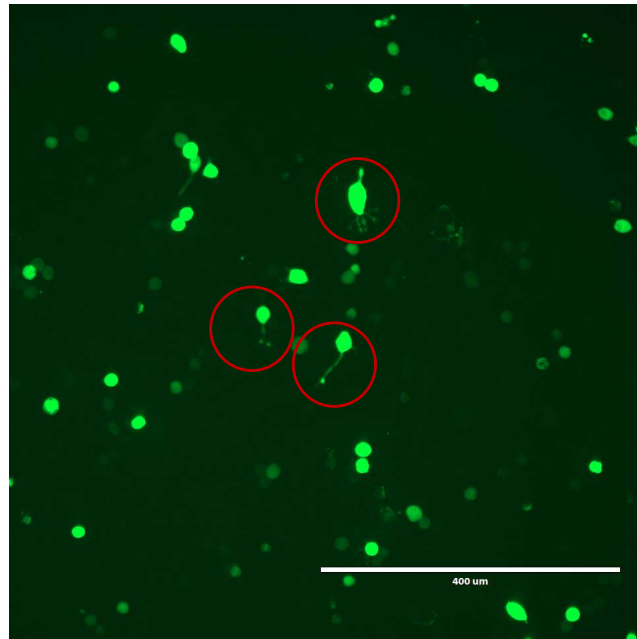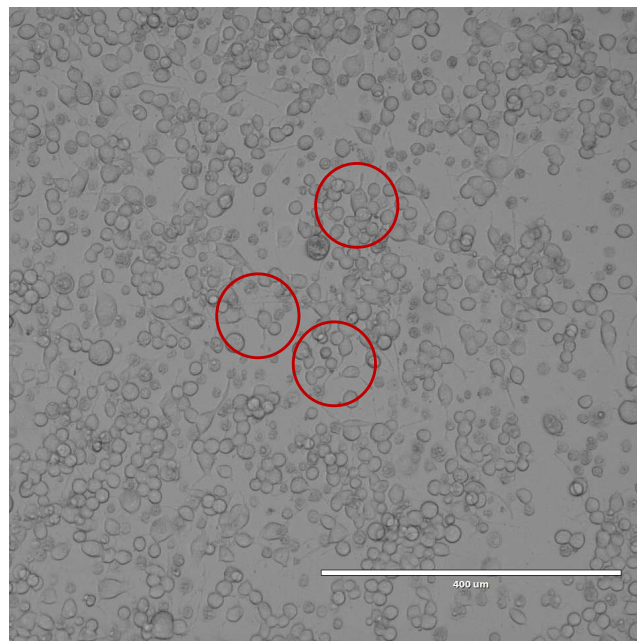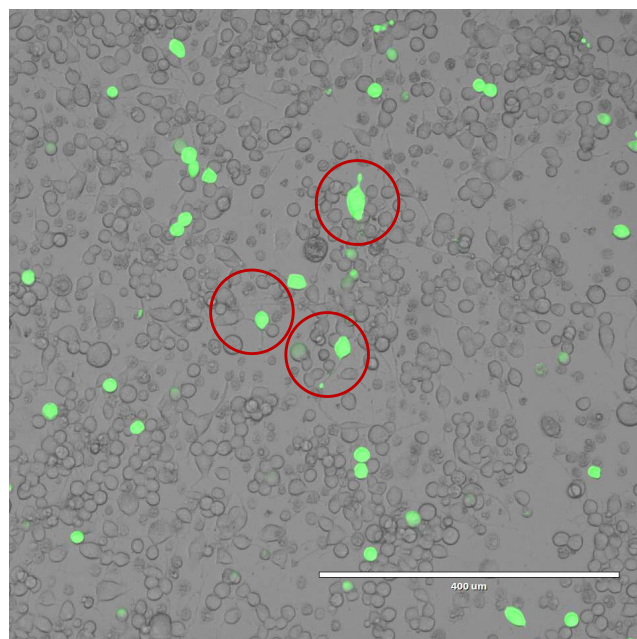

Supplement: Supplementary file 3 — Supplementary Fig.1: Lentiviral ND2 Application Induces Differentiation in Neuro-2a Cells. B) LvND2 group transfected with a NeuroD2 overexpression construct shows clear signs of differentiation. (PDF 269 kb) [file 12017_2025_8852_MOESM3_ESM.pdf]

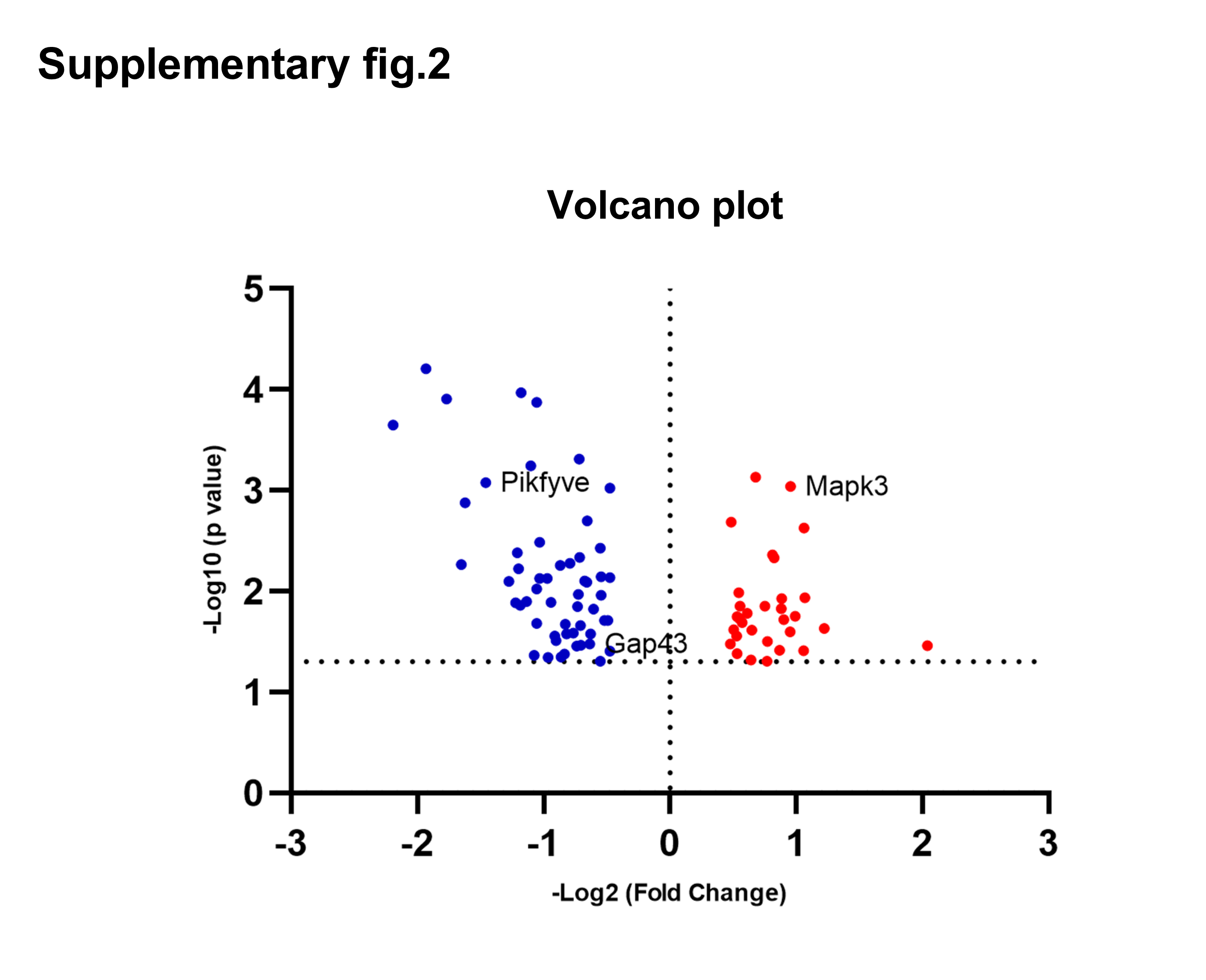

Supplement: Supplementary file 4 — Supplementary Fig.2: Volcano plot. The log twofold change for every protein that was differentially expressed in the LvND2 group was plotted against the negative log 10 p-values calculated using the t-test in a volcano plot. The red color represents elevated proteins in the LvND2 group, while the blue color represents decreased proteins. (TIF 643 kb) [file 12017_2025_8852_MOESM4_ESM.tif]
