# Supplementary material for: Unraveling the Role of NeuroD2 in Ischemic Pathophysiology: Insight into Neuroprotection Mechanisms Associated with AKT Survival Kinase
Source: Neuromolecular Med. 2025 Apr 16;27(1):28. doi: 10.1007/s12017-025-08852-2 (PMC12003519; doi:10.1007/s12017-025-08852-2)
Supplement: Supplementary file 5 — Supplementary Table 1: ND2 Target Genes Involved in the AKT-PI3K Survival Pathway. ND2 binding sites within genomic regions, as identified by Bayam et al., (2015), were analyzed to reveal the top 15 proteins with the highest normalized closest gene scores. These proteins are implicated in the AKT-PI3K survival pathway. (PDF 105 kb) [file 12017_2025_8852_MOESM5_ESM.pdf]

NeuroD2's target genes involved in AKT/PI3K survival pathway

Official Gene symbol	Ensembl Gene ID	Normalized Closest Gene Score	Reference
Akt3	ENSMUST00000111159	26,49581016	
Fgfr1	ENSMUST00000179592	23,38068436	
Creb5	ENSMUST00000047450	21,98628566	
Irs1	ENSMUST00000069799	20,87932404	
Ptk2	ENSMUST00000110036	20,86184638	
Itgb5	ENSMUST00000115028	20,4752129	
Rheb	ENSMUST00000030787	20,41024335	Bayam et al., 2015, Supplementary material
Phlpp1	ENSMUST00000061047	19,93937804	
Itga4	ENSMUST00000099972	19,52435293	
Tlr2	ENSMUST00000029623	19,41252452	
Grb2	ENSMUST00000021090	18,47655882	
Rptor	ENSMUST00000026671	18,15875917	
Itga6	ENSMUST00000133124	17,25400447	
Itgb1	ENSMUST00000090006	17,21363855	
Reln	ENSMUST00000161356	16,58891111	

Supplementary Table 1
